# Abdominal imaging and endoscopic characteristics of adult abdominal IgA vasculitis: a multicenter retrospective study

**DOI:** 10.1080/07853890.2024.2408467

**Published:** 2024-09-26

**Authors:** Yu-Qing Gong, Lin Han, Jin-Yan Zhang, Juan Yu, Na Wu, Wei-Ping Hu, Zhong Xu, Wei Liu, Wei-Feng Huang

**Affiliations:** a Department of Gastroenterology and Hepatology, The First Affiliated Hospital of Xiamen University, School of Medicine, Xiamen University, Xiamen, China; b Department of Gastroenterology, Zhangzhou Affiliated Hospital of Fujian Medical University, Zhangzhou, China; c The School of Clinical Medicine, Fujian Medical University, Fuzhou, China; d Department of Nephrology, The First Affiliated Hospital of Xiamen University, School of Medicine, Xiamen University, Xiamen, China; e Department of Gastroenterology, Yichang Central People’s Hospital, Yichang, China; f Institute of Digestive Disease, China Three Gorges University, Yichang, China

**Keywords:** Adult abdominal IgA vasculitis, computerized tomography, endoscopy, magnetic resonance imaging

## Abstract

**Background:**

IgA vasculitis (IgAV), previously known as Henoch-Schönlein purpura, is an IgA-mediated systemic small vessel vasculitis that tends to be more severe in adults than in children. Early diagnosis of IgAV involving the gastrointestinal tract remains difficult, especially in patients who present with gastrointestinal symptoms before purpura. This study aims to systematically analyze the abdominal imaging and endoscopic features of adult patients with abdominal IgAV, providing assistance to clinicians in the early recognition of this condition.

**Patients and Methods:**

This multicenter retrospective study was conducted in three large tertiary hospitals in China from January 2017 to January 2024. A total of 108 adult patients with abdominal IgAV, who had complete abdominal imaging and/or endoscopy results, were enrolled. The clinical manifestations, abdominal imaging findings, endoscopic characteristics, and serological indicators of the patients were analyzed.

**Results:**

The median age of the patients was 40 years (IQR: 26–55), with a male-to-female ratio of 2:1. Acute abdominal pain was the most common presenting symptom (100 patients, 92.59%). Bowel wall thickening was the most frequent finding on abdominal imaging (50/86 patients, 58.14%). Gastrointestinal endoscopy showed findings of congestion and erosion (32/67 patients, 47.76%), and erosion with ulcers (21/67 patients, 31.34%). Among patients with both imaging and endoscopic results, the duodenum (28/51 patients, 54.90%) and ileum (28/51 patients, 54.90%) were the most commonly affected sites. Laboratory findings revealed elevated white blood cell count (WBC), neutrophil-to-lymphocyte ratio (NLR), C-reactive protein (CRP), D-dimer and fibrinogen levels, along with decreased albumin level. Comparing patients with gastrointestinal symptoms versus purpura as the initial symptom, those with gastrointestinal symptoms had higher levels of WBC (*p* < 0.05) and NLR (*p* < 0.01).

**Conclusions:**

The most common symptom in adult abdominal IgAV patients is acute abdominal pain. In the early stage of the disease, most patients exhibit elevated levels of WBC, NLR, CRP, D-dimer, and fibrinogen, along with decreased albumin level. The duodenum and ileum are the most commonly affected sites. By integrating these findings, clinicians can identify abdominal IgAV patients earlier and more accurately.

## KEY MESSAGES

Adult abdominal IgAV is prevalent in middle-aged adults, with abdominal pain being the main presenting symptom. Abdominal imaging and endoscopy suggest that the duodenum and ileum are particularly susceptible to involvement. Laboratory tests typically show elevated white blood cell count, neutrophil-to-lymphocyte ratio, C-reactive protein, D-dimer and fibrinogen levels, along with decreased albumin level. These findings can aid in the early recognition of IgAV and facilitate timely treatment, thereby improving patient prognosis.

## 1. Introduction

IgA vasculitis (IgAV), formerly known as Henoch-Schönlein purpura, is an IgA-mediated systemic small-vessel vasculitis characterized by palpable purpura, arthralgia or arthritis, and gastrointestinal and renal involvement [1–3]. IgAV involves multiple systems and can be classified into abdominal, articular, renal, and mixed types [4–6]. The incidence of IgAV in children ranges from 3 to 26 cases per 100,000, while it is rarer in adults, with an incidence of 0.1 to 14 cases per 100,000 [3, 7, 8]. Recent studies indicate that adult IgAV symptoms are more severe, particularly with higher gastrointestinal and renal involvement [9, 10]. Notably, gastrointestinal and renal complications are major contributors to poor prognosis and mortality in adult IgAV, with gastrointestinal perforation posing a significant life-threatening risk [3, 11, 12]. Younger patients more commonly experience gastrointestinal and joint involvement, while severe purpura and nephritis are more frequent in older patients [13].

Although IgAV is typically self-limiting, some patients may experience life-threatening visceral involvement [14]. Approximately 48% of adult IgAV patients present with gastrointestinal symptoms at diagnosis, including persistent abdominal pain, nausea, and vomiting [15, 16]. Life-threatening complications such as severe gastrointestinal bleeding and intussusception may also occur during the acute phase of IgAV [17], and studies have also found that patients exhibiting severe gastrointestinal symptoms are more likely to develop severe kidney disease [18]. Endoscopic examination reveals that the descending duodenum and the terminal ileum are the most commonly affected sites, characterized by diffuse mucosal redness, petechiae, erosion, and ulceration [11, 19]. Abdominal computerized tomography (CT) scans typically show segmental thickening of the intestinal wall, predominantly involving the jejunum and ileum, and congestion of mesenteric vessels near the affected intestinal loops [11]. Abdominal CT and endoscopy currently serve as primary diagnostic methods for IgAV with gastrointestinal involvement. Gastrointestinal symptoms typically occur within 8 days of purpura onset, but longer intervals have been reported [20]. In more than 20% of cases, patients experience gastrointestinal symptoms before IgAV develops, posing a considerable diagnostic challenge for clinicians [21]. Case reports even documented that some cases solely exhibit gastrointestinal symptoms without any cutaneous purpura [22–24]. Available studies have found that gastrointestinal symptoms precede purpura in approximately 10–40% of patients, and this is an independent risk factor for intestinal perforation and intussusception, making early identification and treatment of these patients important [25].

The diagnosis of IgAV is based on clinical presentation and histopathologic findings. There are no specific diagnostic laboratory tests [16]. Studies on the early recognition and diagnosis of abdominal IgAV in adults are still relatively scarce. Therefore, exploring the characteristics of adult abdominal IgAV and features of examination findings would facilitate the early recognition of patients with abdominal IgAV.

The aim of this study was to explore the characteristics of abdominal imaging and endoscopic findings in adult abdominal IgAV, as well as to analyze the characteristics of its clinical manifestations and laboratory findings, and to provide clinicians with insights into the early recognition of adult abdominal IgAV by means of these noninvasive examinations.

## 2. Patients and methods

### 2.1. Patients and inclusion criteria

This study was conducted in three comprehensive tertiary hospitals (The First Affiliated Hospital of Xiamen University, Zhangzhou Affiliated Hospital of Fujian Medical University, and Yichang Central People’s Hospital) in China from January 2017 to January 2024. In total, 108 abdominal IgAV patients with complete abdominal imaging and/or endoscopy results were enrolled in the study. The data were collected through the electronic medical records system, which included age, gender, clinical symptoms, CT, Magnetic resonance imaging (MRI), gastroscopy, colonoscopy, and laboratory examinations. Recorded clinical symptoms included abdominal pain, diarrhea, nausea, vomiting, hematochezia, arthralgia and purpura. The distribution of purpura was categorized into three parts: upper extremities, lower extremities, and trunk. The study adhered to the ethical standards of the Helsinki Declaration and obtained an exemption from the ethics committee of each hospital due to the retrospective nature of the study. Due to the retrospective nature of the study and the use of anonymized data, the ethics committee waived the requirement for participant consent.

According to the criteria of the European League Against Rheumatism (EULAR)[26], the diagnosis of IgAV requires the presence of purpura (usually palpable and clustered), or petechiae mainly distributed in the lower extremities without thrombocytopenia or coagulopathy. Additionally, patients were required to exhibit at least one of the following manifestations: abdominal pain (usually diffuse and acute), acute-onset arthritis or arthralgia, renal involvement (hematuria and/or proteinuria), leukocytoclastic vasculitis or proliferative glomerulonephritis, with predominant IgA deposition [27]. Inclusion criteria were as follows: age > 18 years old; diagnosed with IgAV; predominant gastrointestinal symptoms; and complete abdominal imaging and/or endoscopy examinations. Exclusion criteria included: diagnosed with IgAV but without gastrointestinal involvement; presence of coexisting malignancies, viral hepatitis, and hematologic disorders; and missing or incomplete medical records. Gastrointestinal involvement was defined as presence of gastrointestinal manifestations in patients diagnosed with IgAV after excluding other causes of gastrointestinal symptoms. Gastrointestinal manifestations included abdominal pain, nausea and vomiting, diarrhea, intestinal obstruction, intussusception, intestinal bleeding, and intestinal perforation. Renal involvement was defined as hematuria and/or proteinuria. A total of 110 patients meeting the diagnosis of abdominal-type IgAV were screened, with 1 case excluded due to concurrent hematological disease and another case excluded due to missing data, resulting in 108 patients being included in this study.

### 2.2. Imaging and endoscopy

We collected information on abdominal CT and/or MRI findings from 86 patients, as well as endoscopy results from 67 patients. Abdominal CT or MRI was performed in patients who presented with gastrointestinal manifestations such as abdominal pain, and further endoscopy was performed in patients where the CT scan or MRI indicated intestinal wall edema, treatment was ineffective, a diagnosis needed to be confirmed, or there was gastrointestinal bleeding. Among the included patients, the median time for abdominal imaging was 2 (IQR: 1–4) days after admission, and the median time for endoscopy was 4 (IQR: 2–7) days after admission. Abdominal imaging findings were categorized as bowel-wall thickening, lymph node changes, mesenteric changes, intestinal obstruction, intussusception, intestinal stenosis, and normal. Bowel wall thickening was defined as a well-distended lumen with a bowel wall exceeding a thickness of 3 mm. Intestinal obstruction was characterized by bowel dilatation with the presence of intestinal gas and fluid accumulation. Lymph node changes included enlarged or swollen lymph nodes within the retroperitoneal, mesenteric, and pelvic regions. Gastroscopy and colonoscopy findings were categorized as erosive erythema, erosion with ulceration, and normal mucosal appearance. Lesions were analyzed statistically from imaging examinations, endoscopic examinations, and the combination of imaging and endoscopic examinations to determine the affected sites.

### 2.3. Laboratory tests

Laboratory assessments upon admission included white blood cell (WBC), neutrophil-to-lymphocyte ratio (NLR), platelet (PLT), platelet distribution width (PDW), mean platelet volume (MPV), C-reactive protein (CRP), D-dimers, fibrinogen, fibrinogen degradation product (FDP), albumin, creatinine, urea, immunoglobulin A (IgA), immunoglobulin E (IgE), complement component 3 (C3), and urinalysis.

### 2.4. Statistics

Shapiro-Wilk test was performed to assess the normal distribution of continuous variables. Continuous variables with normal distribution were presented as mean ± standard deviation (SD), while those without a normal distribution were presented with median ± interquartile range (IQR). Categorical variables were summarized with frequency counts and percentages. Comparisons between continuous variables were evaluated using t-test or Mann-Whitney U test, while categorical variables were evaluated using chi-square test or Fisher’s exact test. The statistical analysis was conducted with SPSS Statistics 26.0 and GraphPad Prism 9.5.1 software. All tests were two-sided and p-values <0.05 were considered significant.

## 3. Results

### 3.1. Baseline characteristics

A total of 108 adult patients with IgAV who met the inclusion criteria were identified in this study, and their demographic characteristics and clinical manifestations are summarized in Table 1. The median age at diagnosis was 40 years (IQR: 26–55). Among the enrolled patients, 72 (66.67%) were male and 36 (33.33%) were female, yielding a male-to-female ratio of 2:1. Among patients with abdominal IgAV, abdominal pain emerged as the most common gastrointestinal symptom (100 patients, 92.59%), followed by hematochezia (30 patients, 27.78%), nausea or vomiting (25 patients, 23.15%), and diarrhea (22 patients, 20.37%). Purpura was present in 85 patients (78.70%) upon admission, predominantly identified on the lower extremities (81 patients, 98.78%). Additionally, arthralgia was observed in 24 patients (22.22%). Furthermore, the sequence of initial manifestations was recorded in 103 patients. Among these patients, 54 (52.43%) experienced gastrointestinal symptoms before purpura onset, initially presenting with abdominal pain or diarrhea, while 49 (47.85%) had purpura as the initial symptom. Twenty-three patients exhibited only gastrointestinal symptoms on admission, with purpura appearing during hospitalization. In addition, renal involvement was present in 52 out of 102 (50.98%) patients.

**Table 1.** Baseline characteristics and examination features of adult-onset abdominal IgAV (*n* = 108).

Items	N (%)
**Age, median (IQR), years**	40 (26, 55)
**Sex**	
Male	72 (66.67)
Female	36 (33.33)
**Symptom**	
Abdominal pain	100 (92.59)
Purpura	85 (78.70)
Lower limb (*n* = 82)^a^	81 (98.78)
Upper limb (*n* = 82)^a^	39 (47.56)
Abdomen (*n* = 82)^a^	30 (36.59)
Arthralgia / arthritis	24 (22.22)
Hematochezia	30 (27.78)
Nausea or vomiting	25 (23.15)
Diarrhea	22 (20.37)
**First manifestation^b^**	*N* = 103
Gastrointestinal symptom	54 (52.43)
Subcutaneous hemorrhage	49 (47.57)
**Imaging manifestations** ^c^	*N* = 86
Bowel-wall thickening	50 (58.14)
Lymph node changes	32 (37.21)
Mesenteric changes	8 (9.30)
Intestinal obstruction	7 (8.14)
Intussusception	1 (1.17)
Intestinal stenosis	1 (1.17)
Normal	24 (27.91)
**Endoscopy manifestations** ^d^	*N* = 67
Erythema and erosion	32 (47.76)
Erosion and ulceration	21 (31.34)
Normal	17 (25.37)
**Renal involvement (*n* = 102)** ^e^	52 (50.98)

Data are n (%) unless otherwise indicated.

^a^ Missing in three patients.

^b^ Missing in five patients.

^c^ A total of 86 patients underwent abdominal imaging examinations, including CT scans and MRI scans.

^d^ A total of 67 patients underwent gastroscopy and/or colonoscopy examinations.

^e^ Missing in six patients.

### 3.2. Features of imaging and endoscopic findings

The features of imaging and endoscopic findings are shown in Table 1. CT scan and/or MRI scan were performed on 86 patients (79.63%), revealing abnormalities in 62 (72.09%) of these patients. Bowel-wall thickening was the most frequently detected lesion in 50 patients (58.14%), followed by lymph node changes (32 patients, 37.21%), mesenteric changes (8 patients, 9.30%), intestinal obstruction (7 patients, 8.14%), intussusception (1 patient, 1.17%), and intestinal stenosis (1 patient, 1.17%).

**Figure 1.**
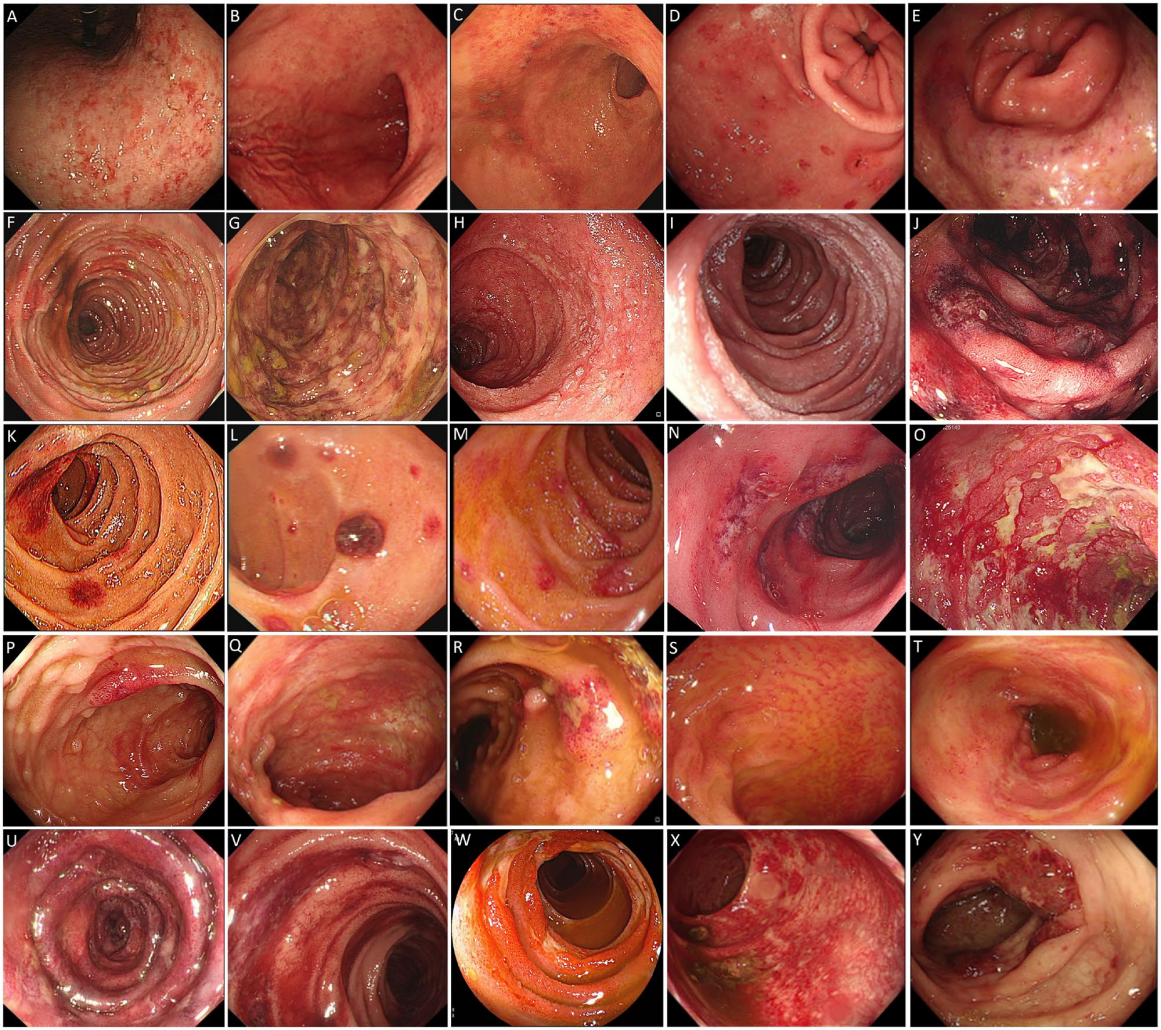
Endoscopic findings in adult abdominal IgAV. (A-E) Stomach: congestion, erythema, and erosion. (F-O) Duodenum: congestion, erythema, erosion, petechiae, and ulceration. (P-Y) Terminal ileum and Colon: congestion, erythema, erosion, petechiae, and ulceration.

Gastroscopy and/or colonoscopy were performed on 67 patients (62.04%), indicating abnormalities in 50 patients (74.63%). The predominant abnormal endoscopic findings included erythema with erosion (32 patients, 47.76%) and erosion with ulceration (21 patients, 31.34%) (Figure 1).

### 3.3. The lesion distributions

The lesion distributions detected by radiographic examinations and endoscopy are detailed in Table 2. Abnormalities were found in 62 patients (72.09%) undergoing abdominal CT or MRI examinations. The ileum was the most commonly affected region (31 patients, 36.05%), followed by the jejunum (19 patients, 22.09%) and duodenum (16 patients, 18.60%). The colon (12 patients, 13.95%), stomach (3 patients, 3.49%), and rectum (2 patients, 2.33%) were also involved. Among patients with duodenum involvement, the descending portion was the most frequently involved region (70 patients, 81.30%).

**Table 2.** The lesion distribution detected by radiographic examinations and endoscopy.

Lesion distribution	N (%)
**Radiographic examinations** ^a^	***N* = 86**
Stomach	3 (3.49)
Duodenum	16 (18.60)
Jejunum	19 (22.09)
Ileum	31 (36.05)
Colon	12 (13.95)
Rectum	2 (2.33)
Normal	24 (27.91)
**Endoscopy**	
**Gastroscopy**	***N* = 61**
Stomach	2 (3.27)
Duodenum	34 (55.74)
Bulb	23 (67.65)
Descending part	24 (70.59)
Horizontal part	6 (17.65)
Normal	25 (40.98)
**Colonoscopy**	***N* = 31**
Ileum	19 (61.29)
Colon	12 (38.71)
Rectum	7 (22.58)
Normal	4 (12.90)
**Combined imaging and endoscopy**	***N* = 51**
Stomach	3 (5.88)
Duodenum	28 (54.90)
Bulb	19 (67.86)
Descending part	20 (71.43)
Horizontal part	6 (21.43)
Jejunum	12 (23.53)
Ileum	28 (54.90)
Colon	15 (29.41)
Rectum	7 (13.73)
Normal	5 (9.80)

^a^ Data from CT scans and MRI scans.

Gastroscopy was performed on 61 (56.48%) patients, showing that 36 (59.02%) had upper gastrointestinal tract involvement. Lesions were predominantly distributed in the duodenum (34 patients, 55.74%), especially the descending portion (24 patients, 70.59%) and the bulb (23 patients, 67.65%), while the horizontal part of the duodenum (6 patients, 17.65%) and stomach (2 patients, 3.27%) were also involved. Colonoscopy was performed on 31 (28.70%) patients, showing that 27 (87.09%) had lower gastrointestinal tract involvement. Lesions were predominantly distributed in the ileum (19 patients, 61.29%), while the colon (12 patients, 38.71%) and rectum (7 patients, 22.58%) were also involved.

Among the 51 patients (47.22%) who underwent both abdominal imaging and endoscopic examination, 46 patients (90.19%) showed abnormalities in either abdominal imaging, endoscopy, or both. In patients with gastrointestinal involvement, the duodenum (28 patients, 54.90%) and the ileum (28 patients, 54.90%) were the most commonly involved regions, followed by the colon, jejunum, and rectum, with incidence rates of 29.41%, 23.53%, and 13.73%, respectively.

### 3.4. The laboratory findings

The laboratory findings of adult-onset abdominal IgAV are presented in Table 3. WBC levels were elevated in 85 (78.70%) patients, with a median of 11.99 (IQR: 9.76–15.51) × 10^9/L. NLR was elevated in 90 (83.33%) patients, with a median of 6.52 (IQR: 4.33–8.94). CRP was elevated in 91 (84.26%) patients, with a median of 20.55 (IQR: 6.57–46.81) mg/L. Albumin was below normal in 73 (67.69%) patients, with a mean of 36.20 ± 0.80 g/L. D-dimer was elevated in 67 (91.78%) patients, with a median of 4.67 (IQR: 1.88–10.05) μg/mL. Only 47 out of 94 (50.00%) patients had elevated fibrinogen, with a median of 4.02 (3.36–4.91) g/L. The mean values of PDW, MPV, as well as the median values of PLT, creatinine, urea, IgE, IgA, and C3, were within normal ranges. Urinalysis showed that 50 out of 102 (49.02%) patients had haematuria, while 59 out of 102 (57.84%) patients had proteinuria.

**Table 3.** Laboratory findings of adult-onset abdominal IgAV (*n* = 108).

Items	
WBC (×10^9/L)	11.99 (9.76–15.51)
WBC > 9.5 × 10^9/L	85 (78.70)
NLR	6.52 (4.33–8.94)
NLR > 3	90 (83.33)
PLT (×10^9/L)	291 (238–367)
PDW (%)	10.50 (8.95–12.05)
MPV (fL)	9.51 ± 0.13
CRP (mg/L)	20.55 (6.57–46.81)
CRP > 3mg/L	91 (84.26)
Albumin (g/L)	36.20 ± 0.80
Albumin < 40g/L	73 (67.69)
Creatinine (μmol/L)	68 (55.90–87.80)
Urea (mmol/L)	5.40 (3.77–6.95)
D-dimers (μg/mL, *n* = 73)^a^	4.67 (1.88–10.05)
D-dimers > 0.5 μg/mL	67 (91.78)
Fibrinogen (g/L, *n* = 94)^b^	4.02 (3.36–4.91)
Fibrinogen > 4g/L	47 (50.00)
FDP (µg/mL, *n* = 94)^b^	11.49 (4.53–39.47)
IgE (IU/ml, *n* = 48)^c^	122.50 (43.10–257.50)
IgA (g/L, *n* = 54)^d^	3 (2.23–3.99)
C3 (g/L, *n* = 60)^e^	1.13 (0.95–1.33)
Haematuria (*n* = 102)^f^	50 (49.02)
Proteinuria (*n* = 102)^f^	59 (57.84)

Data are n (%), mean ± SD, or median (IQR), unless otherwise indicated.

^a^ A total of 73 patients underwent D-dimer testing.

^b^ A total of 94 patients underwent fibrinogen and FDP testing.

^c^ A total of 48 patients underwent Ige testing.

^d^ A total of 54 patients underwent IgA testing.

^e^ A total of 60 patients underwent C3 testing.

^f^ A total of 102 patients underwent urinalysis testing.

Abbreviations: WBC, White blood cell; NLR, Neutrophil-to-Lymphocyte Ratio; PLT, Platelet; PDW, Platelet Distribution Width; MPV, Mean Platelet Volume; CRP, C-Reactive Protein; FDP, fibrinogen degradation products; IgE, immunoglobulin E; IgA, immunoglobulin A; C3, complement component 3.

### 3.5. Comparison of characteristics of different first manifestations

To further explore the features of IgAV patients with gastrointestinal symptoms as the first manifestation, we divided the population into two groups based on the sequence of the appearance of gastrointestinal symptoms and purpura. One group presented with gastrointestinal symptoms first (54 cases) and the other with purpura as the first manifestation (49 cases). The characteristics of different first manifestations are listed in Table 4. The age, gender, abdominal imaging, and endoscopy findings, D-dimer and renal involvement did not exhibit significant differences between the two groups. However, laboratory examination results revealed that patients presenting with gastrointestinal symptoms as the initial manifestation had higher WBC levels [12.11 (10.25–17.80) vs. 11.75 (8.90–13.69), *p* < 0.05] and NLR [7.63 (5.24–13.38) vs. 4.98 (2.97–7.88), *p* < 0.01] compared to those with purpura as the initial manifestation.

**Table 4.** Comparison of imaging, endoscopy and laboratory characteristics of different initial manifestations of abdominal IgAV in adults.

Items	Initial manifestations		P value
	Gastrointestinal symptom (*n* = 54)	Purpura (*n* = 49)	
Age, years, mean ± SD	42.2 ± 17.9	39.7 ± 17.2	0.480
Male	36 (66.67)	32 (65.31)	1.00
**Imaging (*n* = 83)** ^a^	***N* = 46**	***N* = 37**	
Normal	10 (21.74)	14 (37.84)	0.108
Bowel-wall thickening	31 (67.39)	18 (48.65)	0.084
Intestinal obstruction	5 (10.87)	1 (2.70)	0.153
Lymph node changes	16 (34.78)	14 (37.84)	0.773
Mesenteric changes	5 (10.87)	3 (8.11)	0.672
**Endoscopy (*n* = 64)** ^b^	***N* = 35**	***N* = 29**	
Normal	8 (22.86)	9 (31.03)	0.461
Erythema and erosion	17 (48.57)	13 (44.83)	0.765
Erosion and ulceration	14 (40.00)	7 (24.14)	0.179
**Lesion distribution**			
**Gastroscopy (*n* = 59)**	***N* = 34**	***N* = 25**	
Stomach	2 (5.88)	0 (0)	0.217
Duodenum	22 (64.7)	11 (44.00)	0.113
**Colonoscopy (*n* = 29)**	***N* = 17**	***N* = 12**	
Ileum	10 (58.82)	7 (58.33)	0.979
Colon	6 (35.29)	6 (50.00)	0.428
Rectum	3 (17.65)	4 (33.33)	0.403
**Combined imaging and endoscopy (*n* = 49)**	***N* = 29**	***N* = 20**	
Stomach	3 (10.34)	0 (0)	0.138
Duodenum	19 (65.52)	9 (45.00)	0.154
Jejunum	8 (27.59)	4 (20.00)	0.544
Ileum	14 (48.28)	12 (60.00)	0.419
Colon	9 (31.03)	6 (30.00)	0.938
Rectum	3 (10.34)	4 (20.00)	0.342
Normal	2 (6.90)	3 (15.00)	0.357
**Laboratory findings**			
WBC (×10^9/L)	12.11 (10.25–17.80)	11.75 (8.90–13.69)	0.046
NLR	7.63 (5.24–13.38)	4.98 (2.97–7.88)	0.004
CRP (mg/L)	21.43 (6.08–48.10)	18 (8.48–40.48)	0.654
Albumin (g/L)	35.79 (30.02–41.3)	36.49 (32.97–41.38)	0.354
D-dimers (μg/mL, *n* = 70)^c^	5.51 (2.58–14.28)	4.40 (1.42–8.77)	0.280
Fibrinogen (g/L, *n* = 90)^d^	4.36 ± 1.33	4.14 ± 1.22	0.419
Creatinine (μmol/L)	68.00 (53.25–75.00)	72.15 (61.75–80.50)	0.148
**Renal involvement^e^**	***N* = 53**	***N* = 49**	
Yes	26 (49.05)	27 (55.10)	0.541
No	27 (50.94)	22 (44.90)	

Data are n (%) or median (IQR), unless otherwise indicated.

^a^ Data from CT scans and MRI scans.

^b^ Data from gastroscopy and colonoscopy.

^c^ A total of 70 patients underwent D-dimer testing (31 in the gastrointestinal symptom group and 39 in the purpura group).

^d^ A total of 90 patients underwent fibrinogen testing (50 in the gastrointestinal symptom group and 40 in the purpura group).

^e^ A total of 102 patients underwent urinalysis testing (53 in the gastrointestinal symptom group and 49 in the purpura group).

Abbreviations: WBC, White blood cell; NLR, Neutrophil-to-Lymphocyte Ratio; CRP, C-Reactive Protein.

### 3.6. The relationship between D-dimer level and renal involvement

**Figure 2.**
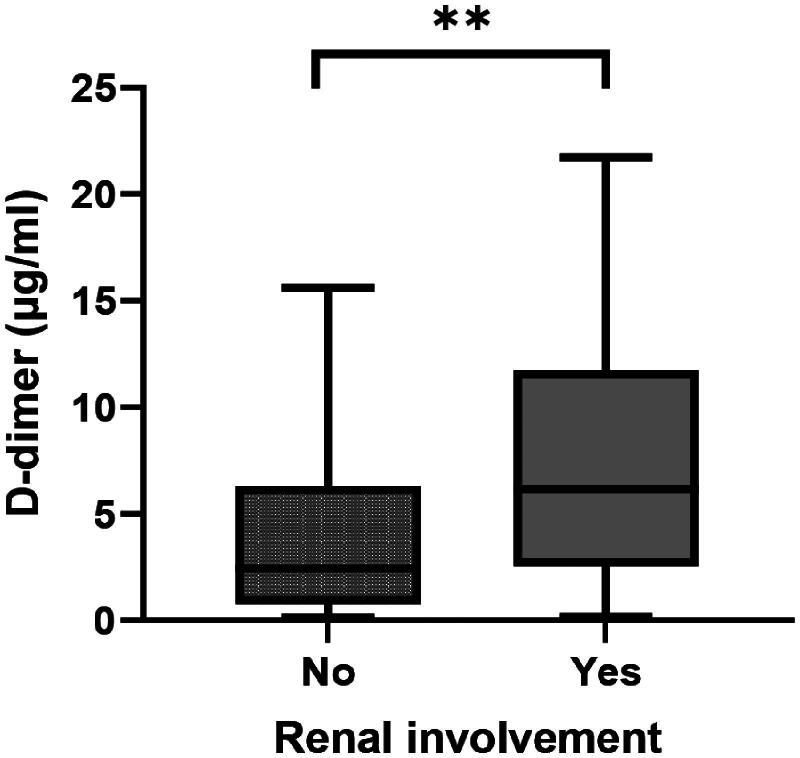
The D-dimer level in patients with and without renal involvement. Patients with renal involvement have higher D-dimer levels than those without renal involvement (*p* < 0.01).

As renal involvement is common in IgAV patients and affects the prognosis, we further investigated the relationship between D-dimer level and renal involvement. The patients were divided into two groups according to whether they had renal involvement. Then, a comparison of D-dimer levels was made. Interestingly, the results showed that D-dimer levels were different between the two groups (*p* < 0.01, Figure 2).

## 4. Discussion

Although IgAV is relatively rare in adults, it tends to be more severe than in children, often leading to severe gastrointestinal complications and an increased risk of developing end-stage renal disease [9]. Therefore, early recognition and treatment are crucial for improving the prognosis of patients with IgAV [16, 28, 29]. Gastrointestinal involvement is common in IgAV, affecting approximately two-thirds of patients [11]. Trapani et al. reported that about 12% of patients exhibited gastrointestinal manifestations as the initial symptom, while 74% presented with purpura [30]. Patients with IgAV typically present with nonspecific gastrointestinal symptoms, including abdominal pain, nausea and vomiting, gastrointestinal bleeding, and diarrhea, which are similar to those of gastrointestinal diseases such as nonspecific gastroenteritis, infectious gastroenteritis, and inflammatory bowel disease [31]. A recent study on adult IgAV revealed that about 24.1% of patients experience gastrointestinal symptoms prior to purpura [32]. Skin palpable purpura is a sign of IgAV, and currently diagnosing IgAV often requires histologic results [8, 14]. However, early recognition of IgAV remains difficult in patients whose gastrointestinal symptoms precede the appearance of skin purpura.

In this study, we focused on abdominal IgAV patients and analyzed the clinical symptoms, abdominal imaging, endoscopic features and laboratory findings to provide insights into the early recognition of IgAV involving the gastrointestinal tract. Adult abdominal IgAV is prevalent in young and middle-aged men, and patients often present with abdominal pain, diarrhea, nausea and vomiting at the time of presentation. These findings are consistent with the frequency of gastrointestinal symptoms documented in prior studies [33]. Purpura was present in 78.70% of patients, with the lower extremity being the most commonly affected site (98.78%).

Abdominal imaging showed that bowel-wall thickening was the most common abnormality (58.14%), with the ileum being the predominant region of involvement (36.05%). Gastroscopy and colonoscopy findings indicated that congestion, edema, erosion, petechiae, and ulceration in the descending duodenum and ileum were the most prevalent presentations. Motohiro Esaki et al. also confirmed the susceptibility of the descending duodenum in patients with IgAV [34]. Among patients who underwent both radiographic examinations and endoscopy, the duodenum (54.90%) and the ileum (54.90%) were identified as the most commonly involved regions. Therefore, further endoscopy can be performed to help identify IgAV in suspicious patients who present with bowel wall thickening and involvement of the ileum, jejunum, and duodenum on abdominal imaging. However, a study of pediatric IgAV using video capsule endoscopy found that the jejunum was the most frequently involved disease location [35]. In our study, imaging results suggested susceptibility of the ileum, jejunum, and duodenum, while endoscopic findings showed involvement of the duodenum and ileum, attributable to the modality of examination. Given the technical limitations, gastroscopy and colonoscopy cannot evaluate the involvement of jejunum and ileum (except for the terminal ileum), whereas CT and MRI imaging can compensate for these limitations. Thus, our analysis of patients with complete gastrointestinal endoscopy and abdominal imaging data indicated that the duodenum and ileum were the most commonly affected sites, aiding in understanding the gastrointestinal involvement lesions of IgAV.

Gastrointestinal involvement in IgAV patients is associated with elevated levels of WBC, D-dimer, and CRP [29, 36, 37]. Damaged vascular walls induced by inflammatory response are common pathological feature of IgAV, leading to hypercoagulation and subsequent thrombolysis. Therefore, elevated D-dimer level can indicate the severity of IgAV. Recent studies have proposed D-dimer as an independent predictor of adult abdominal IgAV and a prognostic indicator for acute gastrointestinal disorders [29, 38]. In order to investigate the significance of D-dimer level for patients’ prognosis, we compared the level of D-dimer in patients with renal involvement and those without, and found that patients with renal involvement had higher level of D-dimer. As renal involvement is a common clinical manifestation in IgAV, associating with the prognosis of patients, it confirms the guiding significance of D-dimer in patient prognosis. Previous studies have also confirmed this finding [29, 39]. The NLR, as an indicator used to assess the systemic inflammatory response, has become a risk factor for various diseases such as cardiovascular diseases, liver cirrhosis, and cancer. Chan et al. have discovered that NLR is capable of predicting gastrointestinal bleeding in adult IgAV patients [2]. In our study, the median value of NLR and D-dimer were elevated compared to normal levels, indicating the diagnostic value of elevated NLR and D-dimer for adult abdominal IgAV. In addition, the mean albumin values were found to be lower than the normal level in this study, which may be attributed to protein loss due to gastrointestinal involvement in IgAV. We also found that the percentage of abnormalities in D-dimer was higher than that of fibrinogen, which suggests the higher sensitivity of D-dimer to IgAV and its greater significance in aiding the diagnosis of IgAV.

We also compared the two groups: those with gastrointestinal symptoms as first manifestation (54 cases) and those with purpura as first manifestation (49 cases). Comparisons revealed that patients presenting with gastrointestinal symptoms as the initial complaint had higher levels of WBC (*p* < 0.05) and NLR (*p* < 0.01) than those presenting with purpura as the initial complaint, warranting further investigation into the potential correlations with patient prognosis.

Based on these findings, we proposed a hypothesis for the early identification of adult patients with abdominal IgAV who first present with gastrointestinal symptoms: In a young-to-middle-aged adult presenting with acute abdominal pain, IgAV should be clinically suspected if laboratory tests indicate elevated WBC, CRP, and D-dimer levels, and abdominal imaging reveals thickening of the duodenum, ileum, and/or jejunum wall. For such patients, timely endoscopy examinations should be performed to assess gastrointestinal involvement and assist in early recognition. Even in the absence of purpura, IgAV should be considered if endoscopic findings are consistent with the common regions and features of the disease. This approach facilitates early recognition and treatment of IgAV, thereby improving patient prognosis.

There are several limitations in the current study. Firstly, as a retrospective and descriptive study, it is susceptible to factors such as missing data and selection bias. Therefore, this study included multi-center data and attempted to minimize the interference of factors such as missing data to improve result accuracy. Secondly, not all patients enrolled in this study underwent both abdominal imaging and gastrointestinal endoscopy, affecting our evaluation of IgAV involvement in the gastrointestinal tract. Additionally, laboratory findings such as IgE and IgA were missing in some patients, although major findings such as WBC, CRP, and D-dimer levels were complete. Furthermore, the hypothesis of early recognition of IgAV proposed in this study is based solely on several qualitative indicators, including WBC, CRP, D-dimer levels, abdominal imaging, and endoscopic findings. We did not establish cut-off values or assign specific scores to each indicator, making it challenging to promote widely in clinical practice and difficult to verify its effectiveness. However, we intend to develop relevant predictive models further and validate the accuracy and reliability of these findings in the future.

## 5. Conclusion

In our study, we found that adult patients with abdominal IgAV exhibited edematous thickening of the ileum, jejunum, and duodenum walls on imaging, as well as erosive erythema and ulceration of the descending duodenum and ileum on endoscopy. These findings suggest that the use of abdominal imaging and endoscopy in young and middle-aged patients presenting with acute abdominal pain and elevated WBC and D-dimer may assist clinicians in early identification of IgAV, enabling early treatment to improve prognosis.

## Data Availability

The datasets generated during and/or analysed during the current study are not publicly available due to including personal medical and life information. But the datasets used and/or analysed during the current study are available from the corresponding author on reasonable request.

## Abbreviations

C3: complement component 3
CRP: C-reactive protein
CT: computerized tomography
FDP: fibrinogen degradation product
IgA: immunoglobulin A
IgAV: IgA vasculitis
IgE: immunoglobulin E
MPV: mean platelet volume
MRI: magnetic resonance imaging
NLR: neutrophil-to-lymphocyte ratio
PDW: platelet distribution width
PLT: platelet
WBC: white blood cell
